# Near-peer education the Leeds way; Gynaecology, Obstetrics and Sexual Health: a case study

**DOI:** 10.15694/mep.2017.000166

**Published:** 2017-09-20

**Authors:** John A W Dalton, Gillian E Lever, Etienne Ciantar

**Affiliations:** 1 Leeds Teaching Hospitals NHS Trust

**Keywords:** Near-peer education

## Abstract

This article was migrated. The article was marked as recommended.

Near-peer education the Leeds way; Gynaecology, Obstetrics and Sexual Health: a case study.

Two doctors in training and a consultant obstetrician present a case study of near-peer education in Gynaecology, Obstetrics and Sexual Health for 4 ^th^ year undergraduate students in Leeds. They offer their own perspectives as near-peer educators, and consider the benefits to medical students, institutions and themselves as near-peer educators.

## Introduction

Near-peer education is now widely accepted in both undergraduate ( Gottlieb et al., 2017) and postgraduate ( Ramani et al., 2016) medical education. Indeed for doctors in training, national regulatory bodies require teaching and supervision of colleagues as a mandatory competency in the UK ( GMC, 2013) and in many other countries ( Ramani et al., 2016).

We therefore seek through this case study, to give an insight into how near-peer education is integrated in Leeds, United Kingdom in both teaching and assessment, specifically in gynaecology, obstetrics and sexual health (GOSH). In Leeds, near-peer education is embraced in both undergraduate teaching and assessment as well as in the teaching of doctors in training for obstetrics and gynaecology. The authors drawn on their own experiences to outline their involvement with near-peer education in terms of giving and receiving near-peer education. In addition, they will highlight the mechanisms which both the University of Leeds School of Medicine and hospitals which receive medical students and doctors in training support and benefit from near-peer education.

In GOSH at Leeds, we have several initiatives where undergraduate medical students are involved as peer-educators, including:

• An undergraduate obstetrics and gynaecology (O&G) breakfast club has been run for several years where undergraduates pick topics and present them to their peers. Pastries are provided and the informal atmosphere is enjoyed by the students.
• A mock Objective Structured Clinical Examination (OSCE) which is run by final year medical students in the Leeds Obstetrics and Gynaecology society (LogSoc) in order to help the fourth year students practise practical OSCE stations and benefits from the experiences of those students who sat the OSCE the year before.

The feedback for these initiatives is excellent and these peer- and near-peer sessions are very well received by the students who attend them. However, we wish to focus on the involvement of doctors in training (junior doctors) as near-peer educators and explore the benefits and challenges associated with it. In particular we will examine the impact this has on the students they teach, the institution as a whole and the impact it has on themselves, as well as what measures are in place in Leeds to provide support to junior doctors as educators. We will focus on undergraduate medical student education for the scope of this paper but peer- and near-peer education also play an important role in postgraduate teaching sessions in O&G.

## ‘Countdown to fourth year’ revision series

Gillian Lever ( **GL**) completed her undergraduate training in the summer of 2016 and is currently a Foundation Year 2 House Officer. As an undergraduate at the University of Leeds she completed an intercalated medical education degree and was President of the Leeds Obstetrics and Gynaecology Society (LogSoc) in her penultimate and final years. During her first year as a qualified doctor she co-ordinated and delivered lectures in a revision series for medical students in their penultimate year (‘Countdown to fourth year’). She will describe her experiences of peer-to-peer and near-peer education, both as an undergraduate and postgraduate.

‘Countdown to fourth year’ is a series of lectures and mock exam questions run during the summer term for students in their penultimate year of medical school. There are eight lectures in total, two of which cover obstetrics, gynaecology and sexual health. Lectures are written and delivered by newly-qualified doctors and based on the current curriculum. Lectures last one hour each and afterwards students are provided with Multiple Choice Questions (MCQs), Extended Matching Questions (EMQs) and medical image questions on the topics discussed to undertake under timed exam conditions. Sessions conclude with a discussion of answers to the mock questions. Paper feedback forms were used to provide lecturers with formal feedback as evidence of teaching for their annual appraisals as new doctors. Students were provided with copies of the lecture slides after the lecture has taken place. They were not provided with the EMQs or MCQs in order to preserve them in a bank of questions for future year groups.

## Undergraduate (4th year) Introductory Week

Etienne Ciantar ( **EC**) is a Consultant in Obstetrics and Medical Education at Leeds Teaching Hospitals NHS Trust and Course Manger for GOSH. He completed a PGCE in 2012 and won a clinical teaching excellence award from the University of Leeds in 2014. He will describe how the teaching is organised for GOSH in the 4 ^th^ year undergraduate curriculum.

Many medical schools have a lecture week at the beginning of each speciality placement to prepare the students for their clinical placements. This continues in Leeds and in GOSH is known as ‘Intro Week.’ Historically this was primarily a series of lectures to all 60 students in the group, with each lecture given by either an academic or a consultant from the teaching hospital, giving insights into their particular subspecialty. This however meant that students sat for a full week of lectures which led to significant fatigue and lack of engagement.

In view of this, a significant overhaul of Intro Week was undertaken in 2011, converting it into small-group workshops. Each day has a particular theme, e.g. low risk pregnancy on the Monday, high-risk pregnancy on Tuesday, Benign Gynaecology (including female and male pelvic examinations using mannequins) on Wednesday etc. Students are divided into groups of 15-20 per workshop and there are three parallel workshops taking place concurrently. This therefore means that the facilitators (usually 1-2 per workshop) deliver their session three times consecutively. However these are interactive sessions, which makes it less tiring for the facilitators and gives the opportunity for all students to participate fully whilst gaining a robust knowledge and skills base prior to their subsequent 5 week clinical placement.

John Dalton ( **JD**) is a trainee doctor in Obstetrics and Gynaecology. He qualified in 2009 and was awarded a National Institute of Health Research (NIHR) Academic Clinical Fellow (ACF) post in 2011 at the beginning of his speciality training (residency). During his three-year ACF post he completed a postgraduate certificate in clinical education (PGCE) and Masters of Education at the University of Leeds. He will describe his experiences as a near-peer educator in both Intro Week teaching and as an examiner for the 4 ^th^ year summative OSCE.

## Undergraduate OSCE

The summative assessment for GOSH takes place at the end of the fourth undergraduate year and is placed among other clinical specialities. Given year groups of between 280 and 300 students, 30-40 examiners need to be recruited on the day of the examination from GOSH specialities alone. This usually requires in the region of 15 registrars (doctors in training) to provide enough cover. In addition, an enthusiastic registrar with an interest in education and assessment is the assessment lead for GOSH and has been for the last few years. JD will discuss his experiences as an examiner and how that has informed his teaching in the discussion section.

## How trainees are supported as educators

While many trainees are enthusiastic about teaching and helping with other duties of the academic department such as careers events and personal tutoring, sometimes trainees may feel anxious if there is not adequate support for them. Certainly, while teaching and feedback skills do feature in the postgraduate O&G curriculum and there are sessions provided to cater for this during their training, trainees may still lack confidence as educators.

### Continuing Professional Development programme

The University of Leeds School of Medicine employs a number of strategies to support and encourage doctors in training as well as NHS consultants (specialists) as educators. It is felt that putting in place the measures which will be discussed below makes those that do get involved feel valued. In addition, they help those who wish to be involved in education develop skills as teachers and assessors. It has been highlighted that trainees feel they need training in teaching skills ( Busari et al., 2002). Leeds Institute of Medical Education has addressed this requirement to ‘train the trainers’ by implementing a Continuing Professional Development Programme (CPD Programme) ‘Workshop programme for medical teachers’ which is extremely popular with trainees and consultants alike and covers a large number of relevant topics including:

• OSCE examiner training
• Small group teaching skills
• Large group teaching skills
• Junior doctors as teachers
• Feedback skills
• Student support
• Teaching using simulation
• Teaching in the ward / clinic

Having attended several of these sessions, JD finds they are extremely helpful in not only gaining teaching and assessment skills and developing confidence; these sessions also help to demonstrate that the University encourages junior doctors to teach and that their participation in undergraduate education is legitimate.

### Red and green card system

The University of Leeds School of Medicine has, for a number of years, provided a system whereby students and staff can offer anonymous feedback on one another, whether a positive comment or minor or serious concern. The system is known as the “Red and green card system.” Students can provide feedback on teachers and mentors who have given excellent teaching during clinical placements. All feedback is sent to the School of Medicine and is then sent out to those who have had red or green cards submitted about them. Green cards come in the form of a certificate of recognition and an email of gratitude including the comments provided by the individual submitting the card.

**GL** discusses her experience of receiving green cards for her bedside teaching:

It demonstrates how seriously the University takes the teaching given by clinicians, including doctors in training. Examples of the comments sent with a green card are shown below:

“ *A really committed doctor who always found time to teach us or explain things in more detail, which really benefitted our learning. Her teaching session was really well thought out and she was very supportive in the clinical environment, which I appreciated*”

*“She has been one of the most engaging and friendly F1 doctors I have come across during my final year placements. She was extremely willing to teach and have us shadow her despite her heavy workload. She helped me to develop practical skills such as prescribing, taking blood cultures and accessing central lines. She deserves a green card for her positive attitude towards students.”*

*“Excellent teaching and making us feel very included at all times”*

**GL:** The green and red card system is valuable to students, staff and NHS organisations as it can highlight exceptional individuals and inspires those in receipt of a green card to continue their good work. It makes those who put the effort into teaching feel valued. Such positive comments demonstrate that not only senior doctors provide beneficial learning experiences. When the NHS is busier than ever before it is often difficult to be sure that, as a teacher, you have made a positive contribution to a student’s learning. The green and red card system is a great way to show appreciation or escalate concerns in a formal and anonymous manner. Green cards can be used by health professionals for annual appraisals as evidence of teaching and positive feedback from students. The above comments highlight the importance of professional role modelling in medical education and we postulate that the nature of near-peer education in the clinical setting helps the students determine their place in the community of practice and feel included as part of the profession. Sometimes perhaps, senior doctors may seem too far detached from life as a senior medical student. It’s almost too difficult to aspire to becoming a consultant when, as a student you’re grappling with the difficulties of venepuncture, cannulation, history taking and examination whereas students can perhaps envisage being a junior doctor.

### Clinical Teaching Excellence Awards

The University of Leeds School of Medicine has an annual ‘Clinical Teachers Day’ to “encourage and reward excellent undergraduate clinical teaching across the NHS in West Yorkshire and Harrogate” by clinical staff. Non-university employees are invited to apply for awards from three different categories:

• Clinical Teaching Excellence Award - for consultants and trainees with more than five years of experience in medical education.
• Clinical Teaching Development Awards - for junior staff who are within the first five years of starting their career in medical education and can show that they have already received positive student feedback and are active in clinical teaching.
• Team Clinical Teaching Excellence Awards - for groups or teams who provide excellent medical education.

The winners of these awards are funded to undertake Postgraduate Certificates in Clinical Education or the Masters in Clinical Education at the University of Leeds. If the winner of the award already holds both qualifications, the University of Leeds will be eligible for a financial award to contribute towards their intended personal and professional development.

JD has been awarded a Development Award and EC has been awarded an Excellence Award. Winning such awards as recognition of the effort and hard work put into teaching is extremely rewarding. However, having the opportunity to undertake postgraduate study in education can really enhance the teaching we can offer.

## Discussion

Through our own perspectives as near-peer educators we will consider the benefits to medical students, institutions (i.e. University and Teaching Hospital Trust) as well as ourselves as near-peer educators.

### Benefits to undergraduate students:

**JD:** In the case of the introductory lecture week where a greater component of teaching was delivered in small groups, it was out of necessity that doctors in training were enlisted to help with the course delivery as well as tutorial provision.

Student feedback for this form of interactive near peer teaching is very encouraging and suggests trainees can be as valuable as consultants in undergraduate education:

“All the teaching during intro week was very engaging, particularly the more practical workshops.”

*“Intro week was pitched at the perfect level with nearly all the relevant information covered.”*

*“Small group sessions during intro week were judged just right. A nice mix of useful topics that didn’t drag on as in some other specialities.”*

**GL discussing the ‘Countdown to fourth year’ session:** Having newly qualified doctors delivering lectures went down well with students. These junior doctors are able to bring practical examples and experience to their teaching which helps to put revision in context for students. As one near-peer educator stated:

“ *It offers positive encouragement for students to learn for life and not solely for the exam.*”

**JD:** I find participating as an OSCE examiner is an extremely developmental experience as it gives me a better insight into what the students have learnt from my teaching sessions. Several years ago while being an OSCE examiner, I got a sense that students knew, for example, how to perform a speculum examination, bimanual examination and swabs, but were perhaps, often unsure of *why* they were doing the examination and which part of the examination they should do at different gestations, for example. This led me to devise a ‘Roundup Lecture’ given to the students at the end of their GOSH placement which covered common clinical presentations and how to manage them through a series of six cases, discussed in an interactive two-hour session. These always get very good feedback and had I not participated as an OSCE examiner, I would not have realised that a session like this would be of benefit. Seeing students perform badly in OSCE stations really makes me reflect on the teaching I give and makes me strive to improve every session I deliver.

### Institutional benefits:

#### EC discusses the impact of trainee involvement as near-peer educators:

Having trainees involved with education reduces the burden on consultants and academic staff in terms of teaching and assessment. Without getting the trainees involved, the small group teaching sessions during Intro Week would be impossible to run. Trainees who are on rotation between different hospitals in the region often bring new and interesting ideas too, which adds a vibrancy to the department. If trainees are recognised by winning an award for example, that raises the profile of the whole department.

#### EC has also noted significant institutional benefits from the introduction of a near-peer education programme in Leeds:

O&G trainees at Leeds Teaching Hospitals have always been exposed to numerous learning opportunities, including informal teaching in clinics and theatre, teaching during perinatal and audit meetings as well as more informal bedside teaching on ward rounds. However the department lacked a robust formal teaching programme. This was picked up in the national GMC trainee survey in which only 35% of O&G trainees were satisfied with the local teaching in 2014, ranking the department a mere 36 ^th^ out of 40 departments in this category.

For this reason a monthly protected teaching programme was set up in 2014. Teaching occurs for half a day and all trainees are expected to attend. All elective obstetrical and gynaecological surgical cases are cancelled, as well as the trainee list in the gynaecology clinics. Antenatal clinics are capped and run exclusively by the consultant when teaching takes place. Consultants and the management team are fully aware of the importance of teaching and are therefore fully supportive of this initiative.

The sessions are themed (urogynaecology, maternal medicine, laparoscopic simulation, etc.) and mapped onto the RCOG postgraduate curriculum. The sessions are conducted by experts in the field, often in association with a doctor in training. One of the sessions is based around post-mortem consenting and also involves trainees observing a neonatal post-mortem.

The programme is coordinated by a senior trainee under EC’s supervision. The introduction of this teaching has led to a marked improvement in trainee satisfaction, with 66% of trainees now being satisfied with the local teaching offered, placing our department in 12 ^th^ place in this domain. This is clearly a marked improvement from a few years ago and we keep working hard to improve this further.

**JD:** We have found that special appointments of trainees to educational roles can help to fill rota gaps which are a problem in many Trusts. In Leeds, we have a post-CCT fellow (a doctor who has completed their speciality training but wants to gain more skills prior to taking a consultant post) in Medical Education. The RCOG offers special options during the final two years of specialty training called Advanced Training Skills Modules (ATSMs), one of which is Medical Education. Successful completion of this module is another accolade which will help the post-CCT fellow obtain a consultant position with an education component.

### Benefits to the trainees themselves:

There is certainly a sense of achievement to be gained from providing near-peer education:

**GL:** Running ‘Countdown to Finals’ was a complex but ultimately rewarding. Prior to organising the lecture series, I had been able to benefit from others’ teaching on the same subjects when I was in my penultimate year of medical school. At the time I remember resolving to volunteer my time to do the same for younger students because I had found it so helpful. When we ran the series I realised that I had not appreciated the magnitude of the job at hand and it took a lot of time to organise. Despite the effort involved, the feedback we received from students made the whole experience extremely fulfilling and I was glad to be able to contribute to the learning of younger students.

**JD:** I gain a great deal of satisfaction and refreshment from teaching the undergraduates. I find it stimulating to teach generally enthusiastic and bright medical students. It’s also a great learning opportunity for me; most of the small group teaching is performed in pairs. The ‘Pelvic Pain and PID session I do jointly with a Genitourinary Medicine trainee, and we learn a lot from each other about recent changes in best practice and gain a better insight into how our specialities interlock. Of course, simply doing the groundwork preparation for teaching also helps to develop your own knowledge and expertise in a subject. Indeed the literature supports the idea that trainees engaging in teaching improves their own clinical skills ( Qureshi et al., 2013).

There are also other, more pragmatic benefits of getting involved as a near-peer educator:

**JD:** Apart from the fact that I find teaching rewarding and stimulating, it is a mandatory requirement as part of our training in O&G to have our Royal College of Obstetricians and Gynaecologists logbook signed off for various types of teaching including small group teaching and lectures, so getting involved with intro week is a great way to fulfil this requirement.

**JD:** In order to fulfil the ‘Observation of Teaching’ requirements for the PGCE I undertook in 2012, it was so helpful to have formal teaching responsibilities where I had a curriculum to deliver which could be observed by the course tutors. Additionally, I think being observed when teaching in a formal setting helped me to improve my teaching sessions. For example, discussing with my tutor how to really nail down clear and concise session objectives has really helped me tailor my sessions to have the biggest educational impact.

**JD:** In addition, having contact with undergraduate students means that even as a trainee, you are often approached by students who want to undertake projects within your speciality. University of Leeds students undertake an ‘Extended Student-led Research or Evaluation Project’ which aims that students completing the course are able to function as consumers and producers of medical research and of clinical service evaluation. While many such projects are supervised by consultants, being a supervisor or co-supervisor as a trainee is a great learning experience in terms of how to supervise students and it means you better able to enlist the help of keen students to assist with one of your own projects. I have also helped supervise students undertake elective projects at a hospital in Uganda I have links with. The students love the experience of medicine in sub-Saharan Africa but by encouraging them to undertake a simple audit project, for example, if it’s of good quality it can culminate in at least a poster presentation which is of benefit for all concerned.

## Conclusion

We hope we have illustrated, through personal experience, the wide-ranging benefits of near-peer education as well as demonstrating through this case study, possible ways to embrace near-peer education within a speciality team. We have shown that junior doctors as educators are essential for the provision of a high quality GOSH programme in Leeds and that feedback from students welcomes the participation of trainees as well as consultants. We anticipate that near-peer education will continue to expand in undergraduate medical education but we feel it is essential that there are mechanisms in place to support and nurture trainees in educational roles to help them perform most effectively.

## Take Home Messages

• Junior doctors can play an important role as near-peer educations in undergraduate teaching and assessment.
• While not a replacement for senior staff, medical students value their contribution to their education.
• It is important to ensure your institution has measures in place to support the training and development of junior doctors as educators.

## Notes On Contributors

Dr John A W Dalton is a Specialty Registrar (trainee) in Obstetrics and Gynaecology at Leeds Teaching Hospitals and an Honorary Lecturer at the Leeds Institute of Medical Education. He conceived and designed the paper and contributed equally to the case study.

Dr Gillian E Lever is a Foundation House Officer Year 2 at Leeds Teaching Hospitlas NHS Trust and contributed equally to the paper.

Dr Etienne Ciantar is a Consultant in Obstetrics and Medical Education at Leeds Teaching Hospitals NHS Trust and Course Manger for Gynaecology, Obstetrics and Sexual Health (GOSH). He contributed equally to the case study.
