# Supplementary material for: Near-peer education the Leeds way; Gynaecology, Obstetrics and Sexual Health: a case study
Source: MedEdPublish (2016). 2017 Sep 20;6:166. [Version 1] doi: 10.15694/mep.2017.000166 (PMC10885296; doi:10.15694/mep.2017.000166)
Supplement: Peer review history

# Reviewer report: Reviewer response for version 1

**DOI:** 10.21956/mep.19351.r28288

**Published:** 2019-04-12

**Authors:** Ken Masters

**Affiliations:** 1 Sultan Qaboos University

This review has been migrated. The reviewer awarded 4 stars out of 5 A really interesting and useful paper showing how medical students can learn from their peers and other students and trainees at a range of different levels. The benefits to both the undergraduates and the junior doctors are nicely elaborated in this case study. One can imagine that the logistics of making this work can be quite demanding, but the case study indicates that the benefits justify the effort.Although every training centre will have differences, I think the paper offer valuable insights for others to use.

Reviewer Expertise:

NA

No decision status is available

# Reviewer report: Reviewer response for version 1

**DOI:** 10.21956/mep.19351.r28287

**Published:** 2017-09-24

**Authors:** Trevor Gibbs

**Affiliations:** 1 AMEE

This review has been migrated. The reviewer awarded 4 stars out of 5 An interesting paper that comes clearly from several faculty with an apparent passion for teaching. Taking this into account it still makes for an interesting read and can provide some sound practical advice to those involved in faculty and curriculum development. A more in depth evaluation is to be encouraged

Reviewer Expertise:

NA

No decision status is available
